# Post-Myocardial Infarction Ventricular Remodeling Biomarkers—The Key Link between Pathophysiology and Clinic

**DOI:** 10.3390/biom10111587

**Published:** 2020-11-23

**Authors:** Maria-Madălina Bostan, Cristian Stătescu, Larisa Anghel, Ionela-Lăcrămioara Șerban, Elena Cojocaru, Radu Sascău

**Affiliations:** 1 Internal Medicine Department, “Grigore T. Popa” University of Medicine and Pharmacy, 700503 Iasi, Romania; madalina_farima@yahoo.com (M.-M.B.); radu.sascau@gmail.com (R.S.); 2 Cardiology Department, Cardiovascular Diseases Institute “Prof. Dr. George I.M.Georgescu”, 700503 Iasi, Romania; 3 Physiology Department, “Grigore T. Popa” University of Medicine and Pharmacy, 700503 Iasi, Romania; ionela.serban@umfiasi.ro; 4 Department of Morphofunctional Sciences I—Pathology, “Grigore T. Popa” University of Medicine and Pharmacy, 700503 Iasi, Romania; elena2.cojocaru@umfiasi.ro

**Keywords:** post-myocardial infarction ventricular remodeling, prognosis, myocardial necrosis biomarkers, neurohormonal activation biomarkers, inflammatory reaction biomarkers, fibrosis biomarkers, apoptosis biomarkers, new generation biomarkers

## Abstract

Studies in recent years have shown increased interest in developing new methods of evaluation, but also in limiting post infarction ventricular remodeling, hoping to improve ventricular function and the further evolution of the patient. This is the point where biomarkers have proven effective in early detection of remodeling phenomena. There are six main processes that promote the remodeling and each of them has specific biomarkers that can be used in predicting the evolution (myocardial necrosis, neurohormonal activation, inflammatory reaction, hypertrophy and fibrosis, apoptosis, mixed processes). Some of the biomarkers such as creatine kinase–myocardial band (CK-MB), troponin, and N-terminal-pro type B natriuretic peptide (NT-proBNP) were so convincing that they immediately found their place in the post infarction patient evaluation protocol. Others that are related to more complex processes such as inflammatory biomarkers, atheroma plaque destabilization biomarkers, and microRNA are still being studied, but the results so far are promising. This article aims to review the markers used so far, but also the existing data on new markers that could be considered, taking into consideration the most important studies that have been conducted so far.

## 1. Introduction

Cardiovascular diseases represent a leading cause of death, accounting for 30% of deaths worldwide. Of these, up to 7 million deaths a year are caused by coronary ischemic disease, accounting for 12.8% of the total. Therefore, the statistics speak of a sad reality: every sixth patient in Europe dies from a heart attack [1]. This explains the increased interest in this pathology and the increased interest that the evaluation of these patients enjoys lately. Increasing access to cardiac catheterization laboratories and, implicitly, to percutaneous myocardial revascularization techniques, has significantly reduced both short-term and long-term mortality in patients with heart attacks.

Therefore, in the context of current knowledge, studies in recent years have shown increased interest in developing new methods of evaluation, but also in limiting post-infarction ventricular remodeling, hoping to improve ventricular function and the further evolution of the patient. This is the point where biomarkers have proven effective in early detection of remodeling phenomena, some of them being so convincing that they immediately found their place in the post-infarction patient evaluation protocol. This article aims to review the markers used so far, and also, the existing data on new markers that could be considered, in order to see the biomarkers that approach the characteristics of an ideal biomarker.

## 2. Ventricular Remodeling—Pathophysiology

Despite this medical progress, in the case of patients who suffered a myocardial infarction, there is more and more concerns about the phenomenon of ventricular remodeling that deeply affects ventricular function and implicitly resonates with the patient’s prognosis. Experimentally, it has been shown that acute ischemia causes important changes in the ventricular architecture, localized changes both in the infarct area and in other segments.

From a physiopathological point of view, the ventricular remodeling manifests under two directions: macroscopic changes that occur after 3 months of onset and microscopic changes that begin from the first moment of the coronary occlusion.

At the macroscopic level, despite complete and successful coronary angioplasty, studies have shown that both in the infarcted area and in the adjacent areas, there is a remodeling process translated into the loss of shortening and contraction with asynchronous abnormalities, hypokinesia, akinesia, and dyskinesia at the level of the ischemic zone and of the initial hyperkinesia followed by the subsequent hypokinesia at the level of the neighboring areas and the final result is a decrease in cardiac pump function, in the cardiac output, and in blood pressure and an increase in ventricular volumes [2,3]. In parallel, the ventricular cavity dilates as a compensatory response to its dysfunction, a process directly related to the magnitude of the infarction area. Its purpose is to maintain a constant beating volume as the percentage of viable contractile myocardium decreases. In the long run, however, this dilation increases the systolic and diastolic parietal stress, thus creating a vicious circle in which the initial dilation generates additional dilation [4].

In addition to myocardial ischemia, at least two other processes participate in this process: the phenomenon of no reflow and the epigenetically mediating disturbance of endogenous repair system.

The no reflow phenomenon is associated with early remodeling and is determined by the microvascular obstruction and dysfunction that disrupts regional perfusion [5]. Studies have shown that the phenomenon of no reflow correlates with the higher incidence of ventricular remodeling and increased risk of cardiovascular events and sudden death [6].

On the other hand, epigenetically mediating disturbance of endogenous repair system translates to altered vascular repair, with maintenance of vasoconstriction and vascular dysfunction in the area adjacent to myocardial infarction [7,8].

At the microscopic level, from the moment of coronary obstruction, a series of nitric oxide disrupting processes are initiated, the vascular signaling systems endothelial growth factors signaling systems are activated, the cytokines are released and this is how the apoptosis and necrosis pathways are activated, generating an increase in oxidative stress, mitochondrial dysfunction, alteration of myocyte metabolism, promotion of fibrosis, and cell remodeling. Therefore, microvascular inflammation, small vessel obstruction, and endothelial dysfunction maintain the remodeling phenomenon [9].

Sequentially, in the first 72 h hours of ischemia, myocytic necrosis appears accompanied by edema and inflammation of the area affected by the infarction. Subsequently, a process of fibroblastic proliferation and collagen storage is installed, which results in the occurrence of the scar. In the period between the resorption of necrotic tissue and scarring, the infarct area undergoes a process of thinning and elongation which is called “infarction expansion” [10]. Proteases and the activation of matrix metalloproteinases (MMPs) released by neutrophils that cause degradation of collagen fibers participate in this process. The final effect is an increased parietal stress which stimulates the mechanoreceptors and generates angiotensin II-releasing intracellular signals. After 72 h, there is a process mediated by the renin–angiotensin–aldosterone system and by neurohormonal activation, which causes changes in ventricular geometry, with dilation of the cavities and myocardial hypertrophy [11,12].

The remodeling process can take from a few weeks to a few months, until a balance between the forces of distension and the resistance offered by the collagen fibers is obtained [1]. This balance is decisively influenced by [13]:

- Characteristics of myocardial infarction: its size, location, and transmurality;
- Extension of the sidereal myocardium;
- Re-permeabilization of the artery responsible for infarction [14];
- Local trophic factors [15].

**Figure 1:**
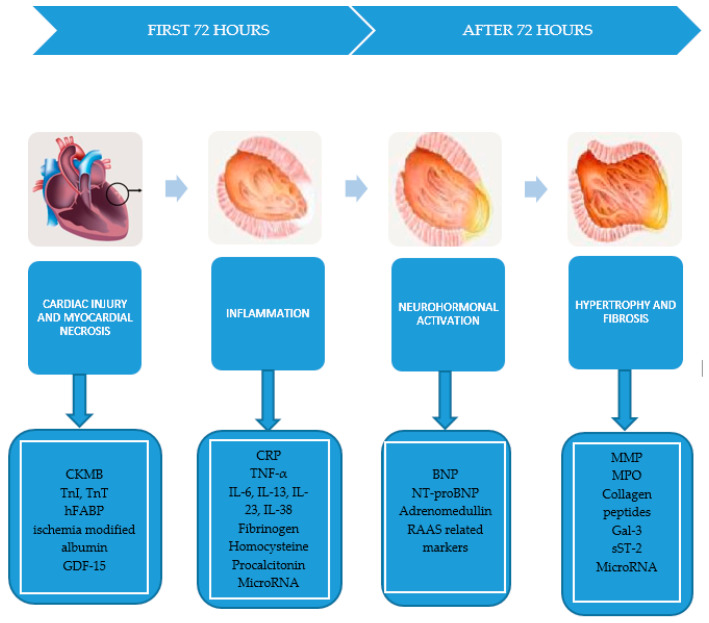
The processes that promote the ventricular remodeling and their specific biomarkers. CK-MB: creatine kinase–myocardial band; hFABP: heart fatty acids binding protein; NT-proBNP: N-terminal-pro type B natriuretic peptide; BNP: type B natriuretic peptide; RAAS: renin–angiotensin–aldosterone system; TNF: tumor necrosis factor; IL: interleukin; MPO: myeloperoxidase; ST-2: suppression of tumorgenicity; GDF-15: growth differentiation factor-15; VEGFR: vascular endothelial growth factor receptor.

In summary, within the ventricular remodeling, four types of processes take place that are closely related to the types of biomarkers that can be detected (Figure 1):

1. Myocardial necrosis: creatine kinase–myocardial band (CK-MB), troponin I and T (TnI, TnT), myoglobin, heart fatty acids binding protein (hFABP), ischemia modified albumin, GDF-15.
2. Neurohormonal activation: N-terminal-pro type B natriuretic peptide (NT-proBNP), type B natriuretic peptide (BNP), adrenomedullin, renin–angiotensin–aldosterone system (RAAS)-related biomarkers.
3. Inflammatory reaction closely related to the release of C-reactive protein (CRP), tumor necrosis factor α (TNF-α), interleukins 6, 13, 23, and 38 (IL-6, IL-13, IL-23, IL-38), homocysteine, procalcitonin.
4. Hypertrophy and fibrosis involving MMP, collagen propeptidases, galectin-3 (Gal-3), soluble ST-2 (sST-2) [5].

There are also some novel biomarkers that are involved in several processes and they cannot be categorized. The main exponents are microRNA (miRNA), which epigenetically regulates the cardiac myocytes apoptosis and increases oxidative stress and inflammation by triggering proinflammatory cytokine release [16,17].

## 3. Biomarkers

The use of biomarkers in the evaluation of patients after acute myocardial infarction has a history of 40 years (the initial term was that of biological marker and was first introduced in 1989), and the scientific trend seems to favor such an approach, which will clearly lead to new studies and new biomarkers.

The characteristics of a biomarker concern three central aspects: the mode of synthesis and release, specificity, and sensitivity [18,19]. The outline of an ideal biomarker is therefore outlined (Table 1).

### 3.1. Biomarkers of Cardiac Injury and Myocardial Necrosis

The first question that arose was whether biomarkers used in the diagnosis of myocardial ischemia could also be interpreted as prognostic markers, so these were the first to be investigated in this respect.

#### 3.1.1. Creatine Kinase MB

CK-MB is an enzyme found in the myocardium, its role being related to the generation of contraction [20]. Its discharge into the circulatory stream is related to myocytolysis and not only to the process of ischemia [21]. It is one of the most used biomarkers in the diagnosis of myocardial injury, being detectable in plasma 4–8 h after the onset of pain and reaching a peak at 18–24 h [20,21]. Studies have placed it above myoglobin in terms of diagnostic value, but recognize its poor specificity in patients with multiple comorbidities such as kidney disease, non-cardiac surgery, chest trauma, muscle disorders, hypothyroidism, hypoventilation, and pulmonary embolism [22,23].

Predictively, studies have shown that a low CK-MB value at the time of diagnosis of AMI means a small amount of affected myocardium and therefore, the success of reperfusion therapy can be maximum, this translating into a lower rate of morbidity and mortality [24]. Clinical data from previous years’ studies have shown the importance of CK-MB at admission as an independent predictor in both the short and long term [25,26,27]. Other studies have shown that not serial CK-MB values, but its increased value for a longer period (values above 124 mg/dL more than 18 h after the onset of myocardial infarction, despite the successful PCI) is correlated with subsequent cardiovascular events (reinfarctions, hospitalizations for cardiac decompensation, death) [28]. Another study was able to correlate the CK-MB peak ratio value (the ratio between the maximum value of CK-MB reached by the patient and the higher value of normal) with a higher mortality at two years post infarction [29]. Some retrospective studies have suggested a correlation between increased CK-MB and long-term mortality [30,31,32,33,34], while others have established that only a significant increase, of 5 to 8 times the upper limit, could have prognostic implications [35,36,37,38]. Yee KC et al. evaluated, in a study, the independent prognostic value of CK-MB in patients with acute coronary syndrome and negative troponin [39] and showed that an increase in CK-MB, even in the absence of troponin dynamics, is correlated with an increase in morbidity and mortality at 6 months. Although these results failed to create a consensus on the use of CK-MB as a prognostic factor, the accessibility and low cost of this analysis could be additional arguments for further studies.

#### 3.1.2. Troponin

Troponin is a protein found in both the heart and skeletal muscles, but I and T isoforms have a higher specificity for the myocardium. This is also the reason why in 2000, the European Society of Cardiology (ESC) and the American College of Cardiology (ACC) introduced in the Universal Definition of Myocardial Infarction, the need for biochemical evidence of myocardial necrosis and indicated as a biomarker of choice, troponin [40]. The sensitivity of myocytolysis detection is significantly higher in the case of troponin as compared to CK-MB, due to a higher percentage of discharge in the circulatory torrent after an acute coronary event, which makes it detectable after a short period of time from the onset of events [41]. Troponin is involved in the binding of actin to myosin and in the regulation of contraction in response to calcium overflow and phosphorylation of contractile proteins. Starting from this mechanism, there was an experimental study that found an inverse correlation of the level of phosphorylation of troponin T dosed in plasma with the risk of ventricular remodeling after acute myocardial infarction [42]. A prospective observational study [43] determined the CK-MB and troponin levels in the first 24 h after onset and correlated them with the evolution of patients one year after the acute coronary event. The results showed that an increase in isolated troponin, in the absence of CK-MB increase, was associated with a higher mortality (6.5% vs. 12.5%), but also in the situation where there was a CK-MB dynamic, the association of increased troponin values led to an increased mortality rate (6.8% vs. 11.7%). In the case of a normal troponin value, in this study, the increase in CK-MB was correlated with a higher mortality, but without statistical significance. Similar data were obtained in a relatively small study conducted in Pakistan that compared the predictive value of creatine kinase with that of troponin T from admission for acute myocardial infarction [44]. They showed that TnT is a better predictor of mortality. Some studies shown that admission troponin is directly related to the incidence of cardiovascular events (cardiac death, non-fatal myocardial infarction, coronary revascularization) and to the mortality rate [45].

#### 3.1.3. Myoglobin

Myoglobin is a heme protein that is found in all types of muscle tissue, but with a higher concentration in the skeletal and myocardial one [46]. This is exactly what makes it a biomarker with low specificity, which is why, at least in the diagnosis of myocardial infarction, the recommendations are to be used in relation to the clinical context, electrocardiography (ECG), and other biomarkers. An important feature, however, is the early growth in plasma (approximately 2 h after the onset of pain), given its small size and high cytoplasmic concentration [47]. However, its sensitivity in the first 2 h after the onset of the acute coronary event is of 70%, which means a good diagnostic performance during this time. It reaches a peak in 6–9 h and disappears from the torrent in the first 24 h [19]. Despite these characteristics, the combined analysis of myoglobin with troponin significantly increased the ability to identify patients with myocardial infarction with increased mortality comparing to either of the two biomarkers evaluated separately [48,49]). Myoglobin is mainly renal eliminated and as kidney disease is a well-known predictor of cardiovascular events, including mortality in patients with a myocardial infarction [50], it has been suggested that the predictive power of myoglobin mortality is due to its ability to identify patients with associated renal failure [51].

#### 3.1.4. Ischemia Modified Albumin

In acute ischemia, the N-terminal end of albumin is damaged, reducing its ability to bind. It has been used in several studies that have shown its usefulness in the diagnosis of acute coronary syndromes in conjunction with tropine values and ECG changes [52]. It has been shown that this combination of biomarkers (troponin and modified albumin) has a predictive value higher than any of them taken separately [53]. However, high values are also found in patients with neoplasms, kidney disease, strokes, and liver disease, which significantly limits its specificity.

#### 3.1.5. hFABP (Heart-Type Fatty Acid Binding Protein)

hFABP is a small protein, located cytosolically, and the role of which is related to the transport and metabolism of fatty acids. The largest amount is found in the myocardium, but we find it in lower percentages in the kidneys, brain, and skeletal muscles [54]. In serum, it appears early after coronary occlusion, at about 30 min, with a peak at 6–8 h and with a return to baseline level after 24–30 h. After 6–8 h from the acute event, its diagnostic value decreases and becomes useless due to accelerated renal clearance [55,56,57]. Studies have also shown an individual predictive value of this biomarker in terms of mortality in patients with acute coronary syndrome [58]. Other studies have hypothesized an even better predictive value than other markers of myocardial necrosis (TnI, CK-MB) for cardiovascular events occurring more than 1 year after an ACS [59]. For patients with chronic heart failure, elevated hFABP levels on admission and discharge were correlated with an increased number of cardiovascular events, including reinfarction and death [60].

#### 3.1.6. GDF-15

Growth differentiation factors (GDF) are a subfamily of proteins belonging to the TGF-beta (transforming growth factor-beta) family. GDF-15 increases with myocardial injury and the inflammation process, suggesting an increased cardiovascular risk [61]. Therefore, studies have shown its increase in myocardial infarction [62] and propose it as an independent predictor of mortality in these patients. Cumulative dosing of TnT/NT-proBNP and GDF-15 has been shown to be very useful in stratifying the risk of these patients [63].

### 3.2. Biomarkers of Neurohormonal Activity

#### 3.2.1. Natriuretic Peptides

BNP is a neurohormone released by myocardial cells following parietal stress associated with the condition of increased intraventricular pressure. As atrial natriuretic peptides, their role is the vasodilation, natriuresis, and inhibition of both the sympathetic nervous system and the renin–angiotensin–aldosterone system [64]. Therefore, both the active form, BNP, and the inactive form, NT-proBNP, can be considered markers of hemodynamic stress. There have been studies that have shown that although these markers may represent predictors for the development of heart failure and death, they do not play an important role as indicators of recurrent infarction [65,66]. Their role in the diagnosis and prognosis of heart failure of any etiology has long been established by extensive studies [67,68]. Regarding their predictive role in patients with myocardial infarction, other studies, such as DETECT, have shown that increased admission levels of NT-proBNP are correlated with higher mortality rates and cardiovascular events at 5 years [69]. Additionally, in these patients, the level of BNP seems to correlate with the size of the myocardial infarction [70]. Their levels at 2–4 days after the acute coronary event may be an independent predictor of left ventricular function and survival after one year [71]. In fibrinolysis-treated infarction, the initial elevated BNP level was correlated with worse reperfusion and 30-day mortality, being considered an independent prognostic factor for mortality, heart failure, and death [72]. There have also been studies comparing the predictive ability of NT-proBNP and BNP with that of TIMI and GRACE scores, with natriuretic peptides proving superior, and their combination with these scores did not significantly increase their predictive value [73,74]. Both BNP and NT-proBNP are therefore excellent biomarkers for cardiovascular events, but their specificity is low, being increased in other forms of heart failure, pulmonary embolism, and kidney damage. Further studies are needed to evaluate their use in various protocols in order to guide the treatment of these patients accordingly to their prognosis.

#### 3.2.2. Adrenomedullin

Adrenomedullin is a regulatory cardiovascular peptide which is increased in the context of the acute coronary event, its role being related to the limitation of infarction and myocardial remodeling. Therefore, although few studies have targeted it, they have shown a role in predicting post infarction remodeling, as well as in stratifying the risk in patients with heart failure and myocardial infarction [75,76].

#### 3.2.3. Renin–Angiotensin–Aldosterone System-Related Biomarkers

RAAS is a hormonal system designed to regulate blood pressure and water balance. After a myocardial infarction, its activation occurs mediated by the increase in ventricular volumes and by vasoconstriction. Aldosterone is associated with a wide range of undesirable effects in the coronary event (endothelial dysfunction, increased oxidative stress, promotion of myocyte necrosis, hypertrophy, and myocardial fibrosis) [51]. Although no other neuropeptide besides BNP and NT-proBNP is routinely used in practical evaluation, there is indirect evidence of their ability to predict morbidity and mortality in patients with infarction by decreasing it in patients treated with RAAS inhibitors [77,78] Some studies have also shown that a higher renin/aldosterone ratio is correlated with higher chances of developing ventricular remodeling [79].

### 3.3. Inflammatory Biomarkers

#### 3.3.1. C-Reactive Protein

This is an acute phase inflammatory protein that causes macrophage activation and is correlated with oxidative stress. The idea of studying it within the pathophysiology of acute myocardial infarction is related to the role of inflammation in atherothrombosis and to CRP synthesis by hepatocytes, as a result of stimulation by inflammatory cytokines, primarily by IL-6. It has long been considered a marker of cardiovascular disease, being correlated with ventricular dysfunction and increased mortality rates among patients with heart failure [80]. Its role in fibrosis and inflammation associated with angiotensin II-induced myocardial remodeling is also known [81]. Some studies tried to recommend CRP as a diagnostic biomarker for myocardial infarction, but low sensitivity and specificity have ruled it out [82]. Studies have shown a direct correlation of CRP dosed at 2 days post PCI with the level of NT-proBNP, infarct size, and ejection fraction and an inverse correlation with non-infarcted myocardial volume, but no association with ventricular volumes was found. The described relationships are observed at 1 week after the acute cardiovascular event, but are lost at 2 months [83]. Similar data were obtained in other studies that managed to correlate CRP not only with infarct size and ejection fraction, but also with the telesystolic volume of the left ventricle measured at admission and at 6 months [84]. Cardiovascular events after an acute myocardial infarction appear to be associated with an initially increased CRP value [85,86,87].

There were also studies that proved the opposite, cancelling by the obtained results, the predictive value of CRP [88]. Its high sensitivity as an indicator of inflammation has been proposed as an independent prognostic marker in patients with acute coronary syndromes [89,90], but without the same ability of troponin to detect patients who may benefit from reperfusion therapy [91,92].

#### 3.3.2. Other Inflammatory Markers

The idea of studying inflammatory markers as predictors for ventricular remodeling after infarction starts from some well-known pathophysiological mechanisms. Coronary heart disease is seen as the product of an inflammatory process. The formation of the atheroma plaque starts from the endothelial injury caused by risk factors (smoking, diabetes, hypertension, dyslipidemia), as it has an important contribution in the process of atherosclerosis. Elevated serum LDL-cholesterol concentrations play a proatherogenic role by stimulating inflammation and oxidative processes in the endothelium. The latter’s response results in the activation of adhesion molecules and the synthesis of inflammatory cytokines [93,94], which thus attracts monocytes and T lymphocytes. The atheroma plaque consists of a lipid center wrapped in a fibrous cap with inflammatory infiltrate. In the development of myocardial infarction, inflammation again plays an important role, the rupture of the plaque triggering a proinflammatory and procoagulant status that ultimately leads to acute thrombotic occlusion. Therefore, it can be stated that inflammation not only promotes the initiation and progression of atherosclerosis, but also contributes to all thrombotic complications [19].

Perhaps the most important inflammatory markers associated with ischemia and reperfusion lesions in acute myocardial infarction are IL-6 and TNF-α. IL-6 is involved in the process of recruitment and activation of inflammatory cells, as well as in CRP synthesis in the liver, having a negative inotropic effect mediated by nitric oxide synthesis [95].

TNF-α is a cytokine with a cardio-inhibitory role that we find in the endothelium, smooth muscle cell, or macrophages and that causes a decrease in myocardial contractility either by direct action or by nitric oxide. There have been studies that have shown the prognostic value in terms of IL-6 mortality in patients with infarction [82,89], while being also able to identify those who could benefit more from an invasive treatment than from a drug treatment [96]. What limits the use of IL-6 as a biomarker for both diagnosis and prognosis is its circadian variation and the small number of studies on this topic [97]. Regarding TNF-α, studies that evaluated its correlation with mortality at 6 months were able to prove its prognostic value together with CRP [98].

Other studies have shown that deficiency of inflammatory factors such as interleukin-13 (IL-13) and interleukin-23 (IL-23) are associated with post infarction ventricular remodeling and a worse long-term prognosis [99,100].

A recent in vitro study showed that interleukin-38 (IL-38) has an increased level of peri-myocardial infarction and that the phenomenon of myocardial remodeling has been markedly improved after the administration of recombinant IL-38. The mechanism involved is related to the decrease in the inflammatory response in dendritic cells [101].

Fibrinogen, an acute phase reactant with direct procoagulant action, is known to be associated with a worse prognosis in the short and long term [87,102,103]. Homocysteine, on the other hand, is associated with the presence of thrombotic material and a greater tendency to reinfarction [104]. However, their individual predictive value is low.

Procalcitonin is a precursor of calcitonin, involved in calcium homeostasis and the synthesis of which is linked to inflammatory processes. There are studies that have shown both its diagnostic value for myocardial infarction [105] and its predictive ability on mortality and the recurrence of ischemic events [106,107].

### 3.4. Biomarkers of Myocardial Fibrosis

#### 3.4.1. Myeloperoxidase

Myeloperoxidase (MPO) is a hemoprotein produced by PMN and macrophages, with a role in converting chlorite and hydrogen peroxide to hypochlorite released in the inflammatory context and involved in the oxidation of LDL-cholesterol particles. This stage is the promoter of foam cell formation in atherosclerosis, which makes MPO a marker of atheroma plaque instability correlated with the risk of developing myocardial infarction in the future. Even if until recently, MPO was thought to be linked only to immune defense [108], recent studies showed its properties as a vascular pro-inflammatory promoter by facilitating the consumption of nitric oxide or by increasing the reactive oxygen species [109]. Particularly, in ventricular remodeling following a myocardial infarction, MPO was proved to increase the collagen deposition in an experimental study that used the ligation of the left anterior descending artery [110] and the MPO-deficient mice exhibited less left ventricular dilatation and attenuated impairment in systolic left ventricular functions [111,112].

Its value increases from the patient with stable coronary heart disease to unstable angina and reaches a maximum value in the patient with infarction [113]. Studies have shown that its diagnostic value is lower than that of the other biomarkers, but elevated values may be independent predictors for cardiovascular events in both acute coronary syndrome patients and healthy individuals [114]. The combined values of MPO, CK-MB, and TnI have shown a more accurate diagnosis of myocardial infarction [115]. A study that evaluated the prognostic capacity of troponin, CRP, and MPO showed that each of them can be used as a biomarker, but the first two had higher values [116].

#### 3.4.2. Metalloproteinases

MMPs are a whole family of endoproteins with many roles in cardiovascular pathophysiology [117], involved in tissue remodeling and degradation of the extracellular matrix and therefore, of collagen, elastin, glycoproteins, proteoglycans, and gelatins. These are controlled by hormonal discharges, growth factors, and cytokines secreted by inflammatory cells and also by tissue inhibitors of metalloproteinase (TIMPs), which are the main regulators for the proteolytic activity [118]. There are four types of TIMPs, three that are present in normal, healthy hearts and one that is more specific to heart diseases [119,120,121,122,123]. Although the main roles of the MMPs and TIMPs are in the extracellular matrix homeostasis, they have also other important functions linked to ventricular remodeling [118,124]. Cardiac fibroblasts (CFBs) can produce a number of MMPs and TIMPs, as a response to the cytokine and chemokines release [125,126,127,128]. TNFα and IL-1β [129], as well as BNP [130], have been reported to induce their production through CFBs. MMPs can also impact on CFBs’ function, as there were studies that have shown that they can trigger fibrosis by cleaving and activating the latent ECM-bound TGFβ, activate the Smad pathway in CFBs, and trigger collagen production [130]. MMP-2 and MMP-9 have particular roles in collagen synthesis [131,132]. Of these, MMP-9 was shown to be correlated with [133].

#### 3.4.3. Collagen Peptides

A 2013 study [134] tried to test a number of markers of fibrosis as elements of post infarction prognosis. Their previous determinations had already shown a correlation of the cardiac extracellular matrix turnover and evolution after the acute coronary event in terms of heart failure development and left ventricle ejection fraction (LVEF) reduction, independent of congestion estimated by using BNP [135]. Prolonging this phenomenon weeks after the infarction increased the risk of decreased LVEF and progression to heart failure, and the combined determination of BNP and TnI after one month refined the prediction of cardiovascular events [100,101,102,103,136,137,138,139]. The study wanted to test the predictive value of collagen peptides dosed at 1 month after infarction. Therefore, they dosed the telopeptide of type I collagen, the aminoterminal propeptide of procollagen type I, and the aminoterminal propeptide of procollagen type III. The results showed that the ratio between type I procollagen aminoterminal propeptide and type III procollagen aminoterminal propeptide over 1, in combination with BNP and LVEF values, may be correlated with a negative prognostic in terms of ventricular remodeling, heart failure, and death.

#### 3.4.4. Galectin-3

Galectin-3 is a lectin that binds to beta-galactosidase. It is secreted by activated macrophages and is involved in cardiac fibrosis, the process of inflammation, and the process of myocardial healing, mechanisms closely related to ventricular remodeling. Increased serum levels in myocardial infarction have long been studied in multiple clinical trials [140,141]. The novelty brought by the latest research is the correlation of Gal-3 with ventricular remodeling and decreased LVEF after myocardial infarction [142]. Additionally, elevated levels of Gal-3 are associated with a higher KILLIP class, hemodynamic instability with intra-aortic balloon pump (IABP) requirements, higher NYHA class, and increased CADILLAC score, and in evolution, these patients are prone to a higher rate of major cardiovascular events, despite effective primary angioplasty [143]. A contradictory result was obtained by Weir et al. [144], which showed the link between galectin-3 and decreased LVEF at 24 months, but without a significant correlation in terms of remodeling per se. In a subgroup of patients, Di Tano et al. showed that in patients with previous myocardial infarction and primary angioplasty, Gal-3 was associated with a higher rate of ventricular remodeling at 1 and 6 months [145], while Gal-3 dosing at 30 days in patients with a first myocardial infarction, treated by angioplasty, showed an increased predictive value in terms of systolic and diastolic ventricular dysfunction [146].

#### 3.4.5. ST2

ST2 is a cardiac biomarker associated with parietal stress and the fibrosis process, with important dynamics in patients with myocardial infarction or acute heart failure [147]. Because of its lack of cardiac specificity, it has been ruled out as a diagnostic tool for myocardial infarction, but other studies have shown promising results on its prognostic value related to mortality and heart failure development for these patients [148,149].

### 3.5. New Generation Biomarkers

#### MicroRNA

**Figure 2:**
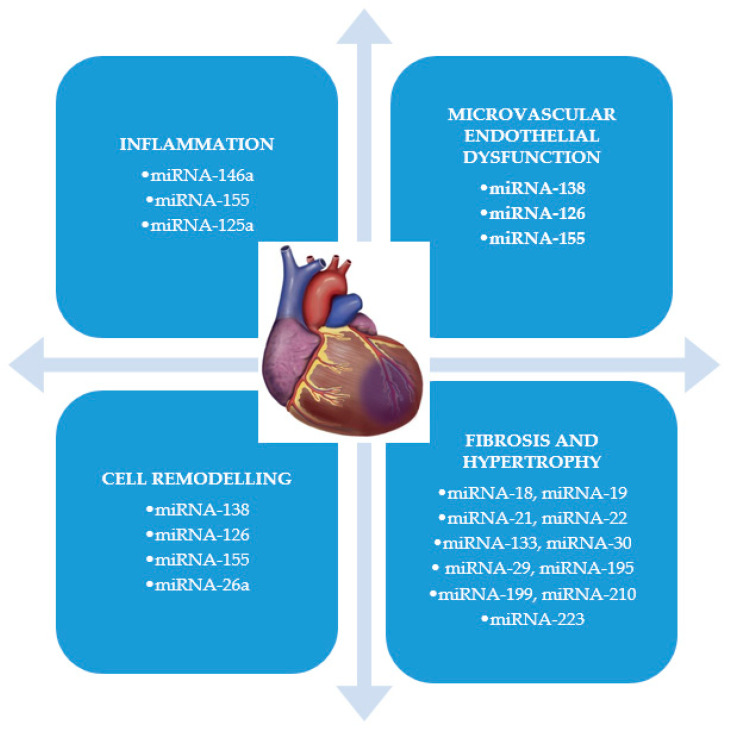
The role of different types of miRNA in the ventricular remodeling. miRNA: microRNA.

MicroRNAs are small RNA molecules without coding function, expressed endogenously, very stable and detectable in plasma, their serum concentration being variable depending on the different pathologies in which these are associated, which makes them suitable as diagnostic or prognostic biomarkers. Studies in recent years have identified multiple cardio-specific microRNAs that appear to play an important role in the development of cardiovascular disease [150] and they have been shown to be linked to almost all the processes that lead to ventricular remodeling [10] (Figure 2). Of these, four appear to be more common in patients with myocardial infarction (miRNA-208a, miRNA-499, miRNA-1, and miRNA-133) [151,152]. Regarding the diagnosis of myocardial infarction, some studies [153,154] indicate as biomarkers miRNA-92 and miRNA-181, while others recommend the combined use of miRNA-1, miRNA-21, and miRNA-499, as having an even higher diagnostic value as hsTnI [155]. Regarding the prognostic value of these biomarkers, miRNA-197a and miRNA-223a were identified as correlated with an increased risk of cardiovascular death [156], while miRNA-134, miRNA-328, miRNA-34a, and miRNA-208b seem to be predictive factors for heart failure development and for an increased risk of post infarction mortality [157,158].

The study conducted by Pin et al. concluded that elevated plasma values of miRNA-208b and miRNA-34a can be considered predictors of left ventricular remodeling after myocardial infarction, associated with higher mortality at 6 months and a 23.1% higher rate of heart failure development. miRNA-208b thus appears to be a cardiac-specific microRNA, with high values in the acute phase of infarction and with a predictive role regarding the development of ventricular dysfunction. Although the miRNA-34 family is considered to have a protective role against pathological remodeling, by overexpression, these prove their ability to induce endothelial cell aging and, implicitly, atherosclerosis [159].

A study led by Devaux et al. [160] found a correlation between miRNA-150 and left ventricular remodeling after a first myocardial infarction. They also showed that miRNA-150, miRNA-101, miRNA-16, and miRNA-27a are linked to a decrease in ventricular contractility.

Some studies have tested the prognostic value of microRNA in patients with primary angioplasty [161]. These identified molecules that are present in plasma even before angioplasty, with rapid dynamics (miRNA-29a, miRNA-29b, miRNA-324, miRNA-208, miRNA-423, miRNA-522, and miRNA-545) and others (miRNA-320a) that are correlated with ventricular remodeling, despite procedural success.

Studies targeting microRNAs have evaluated their prognostic value in terms of two important aspects of post infarction evolution: the ability to predict cardiovascular mortality and left ventricular remodeling.

In terms of mortality, the first molecules identified as having prognostic value were miRNA-133a and miRNA-208b, which were correlated with a significant increase in all-cause mortality at 6 months post infarction [162]. miRNA-208b was studied in other research works too and they identified the same link [151,163]. Subsequently, other microRNA molecules, such as miRNA-499, have been shown to be effective in predicting mortality at 30 days, 4 months, and 1, 2, and 6 years [151,163,164]. Increased levels of miRNA-155 and miRNA-380 have also been shown to be correlated with cardiovascular mortality [165], and miRNA-192, miRNA-194, and miRNA-34 were significantly high in the serum of patients who later developed heart failure [166]. The ability to predict cumulatively both cardiovascular mortality and heart failure development has been attributed to miRNA-145 [167]. A ratio of serum level of miRNA-122-5p/133b measured at the time of cardiac catheterization has also been proposed as a predictor of mortality [168].

In terms of predictive capacity regarding post infarction ventricular remodeling, miRNA-133a has also proven to be a useful tool, being associated with large infarcts with large areas of residual ischemia even after reperfusion [169]. In patients treated with primary angioplasty, increased levels of miRNA-1, miRNA-208b, and miRNA-499 had a negative impact on left ventricular ejection fraction [151]. The same aspect was identified in the case of long chains of RNA lncRNA MALAT1 associated with the decrease in the ejection fraction at 4 months after the infarction [170]. Extensive studies have, in fact, shown the role of long RNA chains in the development of myocardial fibrosis [171,172]. On the other hand, low levels of miRNA-150, miRNA-16, miRNA-27a, and miRNA-101 seem to predict ventricular remodeling [160,173], while increased values of miRNA-208b, miRNA-34a, miRNA-21, and miRNA-155 correlate inversely with the same complication of myocardial infarction [158,174]. Circular microRNA was also not omitted from the studies, as it was associated with left ventricular dysfunction after infarction [175].

Therefore, circulating microRNAs have shown promising results as post-infarction prognostic biomarkers, so other studies should be conducted in order to find a risk stratification formula based on their serum values.

Concluding the results of the previously presented studies, Table 2 presents the prognostic characteristics of each biomarker analyzed in the review, while Table S1 presents detailed data regarding every study protocol.

## 4. Multi Testing

The desire for early ventricular remodeling detection led to the idea of multi-testing, by combining biomarkers generated by different pathophysiological mechanisms. Thus, starting from the premise that TnI, CRP, and BNP are independent markers for post infarction cardiovascular events, a series of studies with promising results were made. Kim et al. [176] tested hsCRP and NT-proBNP, thus showing that the cumulative predictive value is superior to any of them taken separately. At the same time, the use of biomarkers of myocardial stress, inflammation, and myocyte necrosis has increased the predictive capacity for heart failure development [177].

Some studies have even managed to stratify the risk of mortality based on the cumulative dosage of cTnI/CK-MB/myoglobin [178]. Similar data were obtained in patients with STEMI in whom NT-proBNP, hs-TnT, aspartate transaminase (AST), alanine transaminase (ALT), hs-CRP, and lactate-dehydrogenase (LDH) were dosed, showing an increase in their predictive capacity [179]. ST2/GDF-15/hFABP/hs-TnT multi-testing has also shown promising results as a prognostic value [180].

There were also opinions that contradicted the value of multi-testing. Feistritzer et al. [181] showed that the predictive value of hs-cTnT is not improved by adding CK, hs-CRP, LDH, ALT, and AST. Other research has shown that once a biomarker with a high predictive value such as troponin is included in multi-testing, it is difficult to quantify the contribution of other biomarkers added to it.

## 5. Conclusions

The multiple characteristics related to the specificity, sensitivity, early growth, and accessibility that the ideal biomarker should meet have made it difficult to identify a single parameter that meets them all.

Considering the results of our study, we think that the biomarkers that are closest to the characteristics of an ideal biomarker are hsTnI, hsCRP, and NT-proBNP, which have a high level of sensitivity, a high prognostic power, and in addition, the advantage of a low cost and of great accessibility. Out of the desire to refine the prediction, multi-testing was used, which, in most cases, proved to have the specificity and sensitivity of the stronger biomarker, without increasing the power of prediction in this way.

In terms of specificity, fibrosis markers stand out in particular, most having a direct role in the process of ventricular remodeling. The main disadvantage of their use is given by the difficulty of dosing in terms of accessibility and costs, which makes them difficult to use in practice, being reserved especially for clinical trials.

Particular attention must be paid to the new biomarkers; microRNAs that participate in several stages of the ventricular remodeling process are noted as important early markers of remodeling, but also of mortality. We believe that they should be studied in the coming years.

## Figures and Tables

**Table 1:** Characteristics of the ideal biomarker.

Characteristics of the Ideal Biomarker
***High sensitivity***
Increased myocardial concentrations after heart attack
Rapid release to allow early diagnosis
Long half-life to allow late diagnosis
***High specificity***
Its absence in tissues other than the myocardial one
Its absence in healthy patients
***Assay-related characteristics***
Good cost-effectiveness ratio
Easy to assay
Short processing time
High precision
***Clinical characteristics***
Useful in guiding therapy
Useful in predicting the prognosis

**Table 2:** Summarized data about each biomarker’s prognostic value.

Category	Biomarker	Prognostic Value
**Cardiac injury and myocardial necrosis**	CK-MB [25,27,28,29,30,31,32,33,34,35,36,37,38]	Predictive of mortality and cardiovascular events
	Troponin [42,43,44,45]	Independent predictor of ventricular remodeling and cardiovascular events
	Myoglobin [49]	Predictive only in association with troponin
	Ischemia modified albumin [52,53]	Raises the predictive value of troponin when measured together
	hFABP [58,59]	Predictive of mortality and major cardiovascular events after 1 year
	GDF-15 [61,62,63]	Independent predictor of mortality
**Neurohormonal activity**	BNP, NT-proBNP [65,66,69,70,71,72,73]	Highly predictive of heart failure, cardiovascular events, and mortality
	Adrenomedullin [76]	Predictive of cardiovascular events and severity of heart failure
	RAAS-related biomarkers [77,78]	The use of its inhibitors is associated with a mortality and morbidity decrease
**Inflammatory biomarkers**	C-reactive protein [83,84,85,86,87,88]	Predictive of ventricular remodeling and only when associated with other biomarkers, it becomes predictive of mortality
	IL-6 [89,96]	Predictive of mortality and cardiovascular events
	TNF-α [98]	Might be predictive for survival in association with C-reactive protein
	IL-13, IL-23, IL-38, fibrinogen, homocysteine [99,100,101,102,103,104]	Might be predictive of ventricular remodeling
	Procalcitonin [106,107]	Predictive of mortality, cardiovascular events, and ventricular remodeling
**Fibrosis biomarkers**	MMP, MPO [116,133]	Might be predictive of ventricular remodeling
	Collagen peptides [134,135]	Predictive of cardiovascular events and mortality
	Galectin-3 [143,144,146]	Predictive of major cardiovascular events. Might be predictive of ventricular remodeling
	ST-2 [149]	Predictive of survival
**Novel biomarkers**	microRNA [156,157,158,160,162,168,169,170,173,174]	Predictive of mortality, heart failure, cardiovascular events, and ventricular remodeling

CK-MB: creatine kinase–myocardial band; hFABP: heart-type fatty acids binding protein; GDF-15: growth differentiation; RAAS: renin–angiotensin–aldosterone system; IL: interleukin; BNP: brain-type natriuretic peptide; NT-proBNP: N-terminal-prohormone brain-type natriuretic peptide; MPO: myeloperoxidase; MMP: metalloproteinase; TNF-α: tumor necrosis factor α, ST-2: suppression of tumourigenicity-2.

## Supplementary Materials

The following are available online at https://www.mdpi.com/2218-273X/10/11/1587/s1, Table S1: Detailed data of every study protocol.
