# Supplementary material for: Post-Myocardial Infarction Ventricular Remodeling Biomarkers—The Key Link between Pathophysiology and Clinic
Source: Biomolecules. 2020 Nov 23;10(11):1587. doi: 10.3390/biom10111587 (PMC7700609; doi:10.3390/biom10111587)
Supplement: Supplementary file 1 [file biomolecules-10-01587-s001.pdf]

Study	Studied group	Results
Savonitto et al. [25]	11725 patients with ACS for whom the relationship between the maximum value of the CK ratio (the ratio between the CK value and the upper limit of normal) in the first hours after admission and cardiovascular events at 6 months post-infarction was studied	In high-risk patients, even small increases in CK-MB and ratio have independent and important prognostic-related implications.
Szymanski et al. [27]	336 patients with ACS for whom troponin I, CK-MB and myoglobin were measured upon admission with a follow-up of 30 days after the heart attack.	Mortality during hospitalization and within 30 days was higher in patients with significant increases in necrosis biomarkers, being correlated with the number of initially positive markers.
Carvalho et al. [28]	The longitudinal cohort study included patients with acute myocardial infarction followed up to 6 months after the event. All patients received PTCA in the first 12 hours and were assessed for CK-MB levels in relation to cardiovascular events after 30 days and 6 months, respectively (functional capacity, reinfarction, death).	CK-MB has shown its predictiveness related to cardiovascular events at 30 days and 6 months after acute myocardial infarction treated with primary angioplasty.
Cavallini et al. [29]	The study included 3494 patients from 16 Italian tertiary centers, recruited between February 2000 and October 2000.	Postprocedural increases in CK-MB, but not in troponin I, were correlated with higher mortality at 2 years post revascularization.
Abdelmeguid et al. [30]	4484 patients who have undergone successful myocardial revascularization procedures by PTCA.	Even minimal increases of post-PTCA CK-MB levels were correlated with a worse long-term prognosis.
Kong et al. [31]	253 patients who experienced increases in CK and CK-MB after elective PTCA	Increased CK after PTCA has been associated with increased long-term mortality, regardless of clinical variables, severity of coronary heart disease, coronary lesion characteristics, procedural issues.
Akkerhuis et al. [32]	The PURSUIT trial included 9461 patients with ACS, of whom 3778 received revascularization.	Periprocedural myocardial necrosis identified using CK-MB has been associated with increased rates of long-term cardiovascular events.
Ioannidis et al. [33]	The study is a meta-analysis of 7 studies aimed at measuring CK-MB in 23230 patients who received PTCA, as well as correlating its level with the long-term prognosis.	Any increase in CK-MB post PTCA is associated with a statistically significant and clinically significant increase in mortality risk.
Roe et al. [34]	The maximum CK-MB ratio was analyzed in 6164 patients with ACS who underwent PTCA in 4 randomized trials.	Increases in CK-MB and CK-MB ratio are independent predictors of cardiovascular events in the long term.
Kini et al. [35]	A prospective study that included 1675 patients for whom the CK-MB level was measured post PTCA and for whom the cardiovascular events were followed-up during hospitalization and the long-term survival.	Increases in post-PTCA CK-MB have been detected, even in the absence of periprocedural complications, more frequent in patients with diffuse atherosclerosis. Events in hospital occurred mainly in patients with CK-MB increases up to 5 times higher than normal. Medium-term survival was the same for those with elevated CK-MB levels compared to those who did not have any changes.

Baim et al. [36]	A study conducted on 1000 patients with unicoronarian lesion who underwent PTCA	The study concluded that discharging patients with CK-MB increases of 1-5 times the normal value is safe and does not expose them to increased risk of cardiovascular events. No major cardiovascular events (death, reinfarction) were observed in patients with CK-MB increase of up to 3 times the normal value.
Stone et al. [37]	A study on 7147 patients who underwent PTCA and were measured the post-procedural CK-MB levels.	Significant myocardial necrosis (Q-wave on the ECG and CK-MB increases of up to 8 times normal) is a significant prognostic factor for mortality and can be minimized by refining the PTCA technique (type of stent used, atheroablation). For increases smaller than CK-MB, no negative correlation was found with survival.
Brener et al. [38]	The study assesses the impact of increased CK-MB after elective PTCA on major ischemic events in 3478 patients.	There is a clear correlation between myonecrosis and long-term survival.
Yee et al. [39]	The study looked at the prognostic significance of isolated growth of CK-MB in patients with ACS and negative troponin.	The isolated increase in CK-MB is associated with an increased risk of death and major cardiovascular events.
Dubois et al. [42]	Experimental study in mice	There was a significant decrease in serine-phosphorylated TnT and serine-phosphorylated TnT / total TnT ratio in those with intermediate or elevated ventricular remodeling.
Yan et al. [43]	It was a prospective, observational study conducted on 4627 patients with ACS 51 centers participating in the ACS Canadian registration.	The elevated Tn levels were independently associated with worse progression at 1 year, while CK and CK-MB did not provide prognostic correlations.
Kazmi et al. [44]	Prospective study in 186 patients with acute myocardial infarction for whom streptokinase thrombolysis was performed.	The CK value at admission proved to be a better predictor for cardiovascular events, while TnT, for 2-year survival. The combination of the two determinations did not increase their prognostic value.
Matetzky et al. [45]	cTnI was measured upon admission in 110 patients with acute myocardial infarction who underwent primary PTCA.	Elevated values of cTnI upon admission are associated with a higher risk of short-term complications.
McCord et al. [49]	Myoglobin, cTnI and CK-MB were measured at presentation, at 90 minutes, 3 and 9 hours, respectively, in 764 patients with ACS and their individual and combined predictive capacity was analyzed for major events after 30 days (death, reinfarction)	The best predictive value was the combined myoglobin and cTnI measurement at 9 hours.
Mehta et al. [40]; Manini et al. [53]	A prospective study that included patients with ACS whose ischemia altered albumin was measured and major cardiovascular events at 30 days (death, reinfarction) were followed.	The study showed a higher predictive value of albumin, as well as of the measurement in combination with hs-TnT upon admission, compared to hs-TnT measured alone.

Jolly et al.[58]	An analysis targeting 16318 patients with ACS without ST-segment elevation within the GRACE (Global Registry of Acute Coronary Events) registry	hFABP has proven a predictive value which is independent of other biomarkers in predicting mortality in these patients.
Erlikh et al. [59]	203 patients with ACS without ST-segment elevation in whom hFABP, TnI, CK-MB were measured in the first 12 hours after onset.	Among the biomarkers under study, hFABP at 6 hours was the best predictor of major cardiovascular events after 1 year.
Kempf et al. [63]	Circulating GDF-15 levels in 741 STEMI patients included in the ASSENT (Assessment of the Safety and Efficacy of a New Thrombolytic) and ASSENT-PLUS trials were determined.	GDF-15 appears to be an independent predictor of mortality.
Mockel et al. [65]	432 patients with ACS	Even low concentrations of NT-proBNP (≤1400 ng / l) correlate with the higher rate of developing heart failure and death, however without being able to identify patients at risk of reinfarction.
Morrow et al. [66]	1676 patients with ACS without ST segment elevation.	Elevated values of BNP upon admission (>80 pg/ml) identify patients at high risk of death and heart failure and add prognostic value to the individual cTnI measurement.
Leistner et al. [69]	A clinical, longitudinal, prospective trial conducted over 5 years, on 4775 patients who were followed for all-cause and cardiovascular events mortality.	The measuring of NT-proBNP, not of hsCRP, significantly increased the prognostic capacity regarding cardiovascular events.
Niu et al. [70]	A prospective trial on 442 patients with myocardial infarction and PTCA in which the BNP level was measured during hospitalization and after 2 months.	BNP at 2 months proved to be a strong predictor of mortality from any cause and from major cardiovascular events, and the correlation of the initial level with the one after 2 months refined the prediction for all-cause mortality.
Drewniak et al. [71]	The study included 286 patients aged 65-100 years with myocardial infarction, in whom the prognostic significance of NT-proBNP measured on the first day related to one-year survival was assessed.	There was a statistically significant correlation between elevated NT-proBNP levels and the 1-year survival.
Islam et al. [72]	100 patients with acute myocardial infarction in whom the correlation between TnI and BNP levels was studied, without taking into account the clinical manifestations of heart failure.	Elevated TnI was found to correlate with elevated BNP levels and more severe ventricular dysfunction.
Reesukumal et al. [73]	The study included 80 patients with ACS and an average age of 70.68, from whom BNP samples were collected in the first 12 hours after onset. The patients was divided into risk categories according to the TIMI score.	The elevated BNP level was related to a higher mortality at 18 months, without there being a correlation with the TIMI score.
Yuyun et al. [76]	Adrenomedullin was measured in 530 patients admitted with acute cardiac insufficiency, in two large cohorts: Swedish HeArt and bRain failure inVESTigation trial (HARVEST-Malmö) and Italian GREAT Network Rome study.	In both European cohorts, adrenomedullin proved to be a significant prognostic biomarker for the assessment of the severity of the congestion and associated renal impairment, as well as of the worse clinical evolution.

Liu et al. [77]	The study uses data from China Patient-centered Evaluative Assessment of Cardiac Events Retrospective Study of Acute Myocardial Infarction (China PEACE-Retrospective AMI Study) on the use of ACE inhibitors in 2001, 2006 and 2011, and correlates this information with mortality risk data.	There has been a significant decrease in morbidity and mortality in patients with acute myocardial infarction who have received angiotensin converting enzyme inhibitors in their medication.
Pitt et al. [78]	EPHESUS (Eplerenone Post-Acute Myocardial Infarction Heart Failure Efficacy and Survival Study) which analyzed the evolution of patients with acute myocardial infarction and systolic dysfunction (LVEF <40%) who received eplerenone (25-50 mg/day)	The use of antialdosterone in these patients was correlated with a better evolution in terms of morbidity and mortality.
Orn et al. [83]	The relationship between inflammatory markers, myocardial infarction size, post-PTCA ventricular remodeling in patients with a first STEMI and unicoronarian lesion was studied.	The study concludes that the increased level of CRP in these patients is correlated with the dimension of the inflammation phenomenon in the infarction area, thus becoming both a marker and a mediator of myocardial injury.
Schoos et al. [84]	We studied the association of serum complement activation and serum CRP level with changes in telesystolic and telediastolic volumes, as well as with the size of the infarction measured by cardiac MRI after 1-3 days, respectively after 6 months in 55 patients with acute myocardial infarction and primary PTCA.	Complement activation markers were associated with ventricular dilation, and their and CRP's cumulative analysis identified patients with larger areas of infarction and an increased predisposition to remodeling.
Lukin et al. [85]	Clinical, prospective study in 112 patients with ACS and slightly to moderately elevated CRP levels	Major cardiovascular events after 2 years were predicted by hemoglobin, fibrinogen, antithrombin III, cholesterol value, BNP, microalbuminuria. Patients with CRP <3 mg/L showed a clearly higher risk of developing major cardiovascular events after 2 years, if an increase of>50% in the other measured parameters was associated.
Li et al. [86]	A meta-analysis of 13 studies, comprising 1364 patients with ACS in whom CRP levels were studied.	The increased initial levels of CRP are associated with a moderate risk of death and cardiovascular events in the long term.
Fertin et al. [88]	A prospective, multicenter study that included 246 patients at a first STEMI and in whom CRP was measured during hospitalization, after 1, 3 months and 1 year.	No correlation was found with ventricular remodeling.
Cherneva et al. [98]	It included 256 patients with ACS whose TNF-α and hsCRP values were measured during the first 48 hours after admission.	Initially, TNF-α did not correlate with FEVS or myocardial necrosis enzymes. However, HsPCR showed a negative correlation with LVEF and a positive correlation

		with cytolysis enzymes, the latter being a good predictor of 6-month survival.
Hofmann et al. [99]	Experimental study in mice	The genetic deficiency of IL-13 has been associated with worse post-infarction evolution, which is considered to be directly involved in the myocardial healing process.
Savvatis et al. [100]	Experimental study in mice	IL-23 deficiency has led to a more important inflammatory process, with decreased activation of cardiac fibroblasts, phenomena that have led to poor healing and important ventricular remodeling after the infarction.
Wei et al. [101]	Experimental study in mice	Elevated levels of IL-38 have been found in infarction areas, and the recombinant IL-38 injection has significantly improved ventricular remodeling.
Toss et al. [102]	The study included 965 patients with ACS in whom the link between baseline fibrinogen levels and CRP was analyzed after a 5-month evolution.	Elevated levels of both fibrinogen and CRP have been associated with poorer evolution, each with independent predictive value.
Sanchis et al. [103]	385 patients with ACS were studied.	Acute phase reactants were correlated with post-infarction progression, regardless of clinical variables, risk level or troponin value.
Bozkurt et al. [104]	A study was performed on 61 patients at a first STEMI, who underwent fibrinolytic therapy in the first 12 hours and then coronary angiography in the first 72 hours.	An inverse relationship has been shown between serum homocysteine levels, the culprit vessel patency and the flow into the affected coronary artery.
Ataoğlu et al. [106]	It included 77 patients with ACS in whom procalcitonin levels were measured initially and then 48 hours post-admission and they were followed during the first 6 months.	Elevated procalcitonin levels at 48 hours were associated with more inflammatory status and higher mortality in both the acute and 6-month phases.
Kelly et al. [107]	Major cardiovascular events were followed up after 2 years in 977 patients with acute myocardial infarction in whom procalcitonin was measured initially.	A correlation has been shown between procalcitonin levels, cardiovascular events, ventricular dysfunction and post-infarction remodeling.
Urbano-Moral1 et al. [116]	It included 112 patients with STEMI and primary PTCA who were under follow-up for the first 6 months.	Maximum elevated TnT, matrix metalloproteinase 9, and hsCRP values were independent predictors of ventricular remodeling.
Eschalier et al. [134]	A prospective, multicenter study that included 246 patients at a first STEMI.	The low amino terminal propertied ratio of type III procollagen/type 1 collagen telopeptide (≤1) ratio one month after STEMI had predictive value, and the cumulation with the BNP and LVEF levels increased the predictive capacity on mortality and hospitalizations for heart failure.
Iraqi et al. [135]	A sub-study of EPHESUS that measured collagen levels in 476 patients with acute myocardial infarction, ventricular dysfunction and congestive heart failure.	Elevated levels of type I collagen telopeptides and BNP are associated with increased rates of major cardiovascular events and can be ameliorated by eplerenone in the acute phase.
Tsai et al. [143]	The study was conducted in 196 patients with STEMI and primary PTCA in whom circulating galectin-3 levels were measured.	Elevated levels of galectin-3 have been associated with a higher rate of major cardiovascular events in 30 days.

Weir et al. [144]	Galectin-3 was measured 48 hours and 24 weeks post-infarction in 100 patients with STEMI and MRI-evaluated ventricular dysfunction.	Galectin-3 has been associated with increases in extracellular turnover biomarkers, but no correlation has been shown with ventricular remodeling.
Andrejic et al. [146]	The study was conducted on 57 patients with a first STEMI where galectin-3 was measured on the first day and 30 days after infarction, in order to find the correlation with ventricular remodeling after an acute event.	Galectin-3 levels at 30 days have been shown to be an independent predictive factor for 6-month remodeling, and it correlated positively with echocardiographic parameters.
Zhang et al. [149]	It was conducted on 59 patients with acute myocardial infarction.	Serum ST-2 levels have been shown to be a predictive factor independent of other variables and it can be combined with NT-proBNP levels and GRACE score. Both the ST-2 and the IL-33 / sST2 ratio were correlated with 6-month survival.
He et al. [157]	Circulating levels of miRNA-328 and miRNA-134 were measured in 359 patients with acute myocardial infarction.	Elevated miRNA levels have been associated with an increased risk of mortality and heart failure at 6 months.
Schulte et al. [156]	Circulating levels of miRNA-126, miRNA-197 and miRNA-223 were measured in 873 patients with acute myocardial infarction.	Elevated circulating levels of miRNA-197 and miRNA-223 have been identified as predictors of mortality in these patients.
Lv et al. [158]	It included 359 patients followed up for 6 months after the acute myocardial infarction.	Circulating levels of miRNA-208b and miRNA-34a may be considered predictive factors for ventricular remodeling, heart failure, and mortality.
Devaux et al. [160]	The study was conducted in 90 patients with a first STEMI, followed up during the first 6 months.	Low levels of circulating miRNA-150 were associated with significant ventricular remodeling.
Widera et al. [162]	It included 444 patients with ACS followed up in the first 6 months relating to all-cause mortality.	miRNA-133a and miRNA-208b were independent predictors of mortality, independent of age and sex variables.
Gidlof et al. [163]	miRNA levels (miRNA-1, miRNA-208b and miRNA-499-5p) were measured in 424 patients with ACS.	They were correlated with the severity of ventricular dysfunction and the risk of death and heart failure.
Dong et al. [168]	miRNA-145, NT-proBNP, CK-MB and TnI levels were measured in 246 patients with a first STEMI and primary PTCA	Circulating levels of miRNA-145 correlated independently with major long-term cardiovascular events.
Cortez-Diaz et al. [169]	The study included 142 patients with STEMI and primary PTCA, where miRNA-1-3p, -122-5p, -133a-3p, -133b, -208b-3p and -499a-5p were measured at the time of angioplasty and they correlated with acute and long-term events.	The miRNA-122-5p / 133b ratio has been shown to be a new prognostic marker for major cardiovascular events.
Vausort et al. [171]	The study included 414 patients with STEMI and primary PTCA, iin which levels of 5 lncRNAs were measured.	The level of lncRNAs has shown prognostic value for ventricular remodeling.
Devaux et al. [175]	The study included 150 patients with acute myocardial infarction in whom miRNA-16, miRNA-27a, miRNA-101 and miRNA-150 were dosed and they were evaluated echocardiographically after 6 months.	Patients with low levels of miRNA-150 and miRNA-101 and elevated levels of miRNA-16 and miRNA-27a were at high risk of developing ventricular dysfunction.

Liu et al. [177]	Circulating levels of miRNA-146a and miRNA-21 were measured in 198 patients with STEMI and primary PTCA, who were then followed up echocardiographically after 5 days and 1 year.	miRNA-146a and miRNA-21 values were higher in patients with ventricular remodeling, being considered independent predictive factors.
Kim et al. [180]	Initial levels of hs-CRP, NT-proBNP and Tn-I were measured in 215 patients with ACS.	The combined analysis of the 3 biomarkers brought a further prognosis in the risk stratification regarding these patients.
O'Donoghue et al. [181]	Biomarkers representing different pathophysiological axes were used in 1258 patients in the CLARITY-TIMI 28 trial (Clopidogrel as Adjunctive Reperfusion Therapy-Thrombolysis in Myocardial Infarction 28).	In these patients, the multimarker strategy added value to predicting death and development of heart failure at 30 days.
Schernthaner et al. [184]	Levels of sST2, GDF-15, soluble urokinase plasminogen activator receptor, hFABP and plasma fetuin A were measured in patients with acute myocardial infarction.	They were correlated both with the evolution of patients (LVEF, hospitalization period) and with the markers of myocardial necrosis.
Feistritzer et al. [185]	The study included 128 patients with STEMI evaluated under MRI in the first week and where hs-TnT, CK, NT-proBNP, hs-CRP, LDH, AST and ALT were measured.	hs-TnT, CK, hs-CRP, LDH, AST and ALT provided the same prognostic value regarding ventricular remodeling. The prognostic value of NT-proBNP peak concentrations was lower. The study concluded that the multimarker approach did not increase the independent predictive value of hs-TnT.
Reinstadler et al. [183]	NT-proBNP, hs-TnT, AST, ALT, LDH and hs-CRP were measured in 123 patients with STEMI and primary PTCA.	The combined measuring of NT-proBNP, hs-TnT, AST, ALT, hs-CRP and LDH showed higher predictive capacity compared to each biomarker taken separately.

CK-MB: creatine kinase-myocardial band; CK: creatine kinase; ACS: acute coronary syndrome; PTCA: percutaneous transluminal coronary angioplasty; ECG: electrocardiogram; TnT: troponin T; cTnI: cardiac troponin I; hs-TnT: high-sensitive troponin T; hFABP: heart-type fatty acid binding protein; GDF-15: growth differentiation factor-15; STEMI: myocardial infarction with ST-segment elevation; NT-proBNP: N-terminal-pro hormone brain-type natriuretic peptide; BNP: brain-type natriuretic peptide; hsCRP: high-sensitivity C-reactive protein; TIMI score: thrombolysis in myocardial infarction; RASS: renin-angiotensin-aldosterone system; LVEF: left ventricular ejection fraction; CRP: C-reactive protein; MRI: magnetic resonance imaging; TNF- α: tumor necrosis factor α; IL-13: interleukin 13; IL-23: interleukin 23; IL-38: interleukin 38; MPO: myeloperoxidase; ST-2: soluble supression of tumourigenicity -2; miRNA: micro RNA; sST2: soluble suppression of tumorigenicity-2; GDF-15: growth-differentiation factor-15; LDH: lactate dehydrogenase; AST: aspartate transaminase; ALT: alanine transaminase.
